# Assessment of HIV viral load monitoring in remote settings in Vietnam - comparing people who inject drugs to the other patients

**DOI:** 10.1371/journal.pone.0281857

**Published:** 2023-02-21

**Authors:** Louise H. Lefrancois, Binh Thanh Nguyen, Tram Thi Phuong Pham, Nhung Thi Hong Le, Huyen Thi Thanh Dao, Tram Hong Tran, Khanh Phuong Ngo, Ha Thi Tong, Huong Thi Thu Phan, Mohand Ait-Ahmed, Thang Hong Pham, Tuan Anh Nguyen, Fabien Taieb, Yoann Madec, for the MOVIDA 2 study group

**Affiliations:** 1 Epidemiology of Emerging Diseases, Institut Pasteur, Université de Paris, Paris, France; 2 National Reference Laboratory of HIV Molecular Biology, National Institute of Hygiene and Epidemiology, Hanoi, Vietnam; 3 Training and Research Management Center, National Institute of Hygiene and Epidemiology, Hanoi, Vietnam; 4 Vietnam Administration of HIV/AIDS Control, Ministry of Health, Hanoi, Vietnam; 5 Center for Translational Research, Institut Pasteur, Université de Paris, Paris, France; 6 HIV/AIDS Department, National Institute of Hygiene and Epidemiology, Hanoi, Vietnam; 7 Department of International Affairs, Institut Pasteur, Université de Paris, Paris, France; University of North Carolina at Chapel Hill, UNITED STATES

## Abstract

**Introduction:**

Increasing access to viral load (VL) monitoring is essential to fight HIV epidemics. In remote settings in Vietnam, using dried blood spot (DBS) sampling for specimen collection could improve the situation. Here, people who inject drugs (PWID) represent many newly antiretroviral therapy (ART)-initiated patients. The goals of this evaluation were to evaluate if access to VL monitoring and the rate of virological failure differed between PWID and non-PWID.

**Methods:**

Prospective cohort study of patients newly initiated on ART in remote settings in Vietnam. DBS coverage at 6, 12 and 24 months of ART was investigated. Factors associated with DBS coverage were identified through logistic regression, as were factors associated with virological failure (VL ≥1,000 copies/mL) at 6, 12 and 24 months of ART.

**Results:**

Overall 578 patients were enrolled in the cohort, of whom 261 (45%) were PWID. DBS coverage improved from 74.7% to 82.9% between 6 and 24 months of ART (p = 0.001). PWID status was not associated with DBS coverage (p = 0.74), but DBS coverage was lower in patients who were late to clinical visits and in those in WHO stage 4 (p = 0.023 and p = 0.001, respectively). The virological failure rate decreased from 15.8% to 6.6% between 6 and 24 months of ART (p<0.001). In multivariate analysis, PWID were more at risk of failure (p = 0.001), as were patients who were late to clinical visits (p<0.001) and not fully adherent (p<0.001).

**Conclusions:**

Despite training and simple procedures, DBS coverage was not perfect. DBS coverage was not associated with PWID status. Close management is required for effective routine HIV VL monitoring. PWID were more at risk of failure, as were patients who were not fully adherent and patients who were late to clinical visits. Specific interventions targeting these patients are needed to improve their outcomes. Overall, efforts in coordination and communication are essential to improve global HIV care.

**Trial registration:**

Clinical Trial Number: NCT03249493.

## Introduction

In one decade, antiretroviral therapy (ART) coverage greatly increased from 7.8 million people in 2010 to 27.4 million (73%) in 2020 [1]. The increase in ART coverage is essentially explained by the relaxed WHO clinical and immunological conditions for ART initiation over the last few years, eventually recommending to treat all people living with HIV (PLHIV), whatever their clinical and immunological status [2]. Access to HIV viral load (VL) monitoring remains a major issue, especially in remote settings. In its 2016 guidelines, the WHO recommended using dried blood spots (DBS) where plasma was not feasible [2]. DBS has the advantages of being easy to collect, maintain as RNA is stable over long periods of time, and transfer at ambient temperature by regular postal services because, unlike plasma, it is not considered a biohazard. Several studies have evaluated and compared HIV VL results obtained on DBS and on plasma with high sensitivity and specificity, exceeding WHO requirements [3–6]. In addition, some studies have demonstrated the ability of DBS to reach people in remote settings in Africa and Asia [7, 8].

In Vietnam, HIV prevalence in adults was 0.3% in 2019, but much higher in some key populations such as people who inject drugs (PWID) in whom it was 12.7% [9]. To help make routine HIV VL testing available in remote settings, the MOVIDA (Monitoring Of Viral load In Decentralised Area) 2 study was implemented in six remote provinces in North Vietnam. We have already documented the feasibility of DBS for HIV VL monitoring at 6 months of ART in a setting where PWID account for nearly 50% of newly ART-initiated patients [8]. It has been shown that PWID suffer from stigmatization [10, 11], and one can wonder if access to HIV care and response to ART in PWID still differ from those in non-PWID.

This study aimed at describing different outcomes of HIV VL monitoring in HIV infected adults on ART in remote settings and evaluating if PWID have worse outcomes than the other patients (i.e. non-PWID). The goals were i) to describe DBS coverage for HIV VL monitoring; ii) evaluate the rate of virological success at 6, 12 and 24 months of ART and compare it between PWID and non-PWID, iii) evaluate use of the HIV VL monitoring algorithm in terms of availability of confirmatory DBS after failure.

## Methods

MOVIDA 2 is a prospective, observational, multicenter cohort study that aims to improve access to HIV VL monitoring, introducing the use of DBS, in HIV-infected adults followed in remote settings in Vietnam [8]. It was implemented in 43 HIV outpatient clinics (OPCs) across six remote provinces in North Vietnam (Lai Chau, Lao Cai, Phu Tho, Thai Nguyen, Thanh Hoa and Yen Bai) where no laboratory able to perform HIV viral load testing was available and where no system for the routine transfer of plasma samples was implemented. The clinical sites, where the study was conducted, were scattered within these provinces. It must be noted that these provinces are characterized by a mountainous geography, further isolating some clinical sites. From June 2017 to April 2018, all HIV-infected adults (age ≥18 years) who initiated ART, while ART-naïve, were invited to participate in the MOVIDA 2 study; no other selection criteria were applied. Within MOVIDA 2, follow-up of each participant started at ART initiation and continued until the last enrolled patient reached the virological evaluation at 24 months, data were therefore collected from June 2017 to July 2020.

The MOVIDA 2 study being observational, follow-up and care complied with national guidelines. Following the WHO [2], national guidelines recommended initiating ART with a combination of two nucleotide reverse transcriptase inhibitors and one non-nucleotide reverse transcriptase inhibitor. Patients attended weekly visits the first month, and then monthly visits, or bi-monthly if in stable health. The only intervention was the use of DBS for blood sampling. Most demographic data were directly retrieved from the medical file, while data related to ethnicity, distance to care site, professional activity were obtained from the patient at enrolment. Clinical data were retrieved from the medical file.

For HIV VL monitoring, the national algorithm, based on WHO guidelines, recommended HIV VL testing at 6 months of ART, 12 months of ART and then every 12 months afterward. At these timepoints, if the HIV VL was ≥1,000 copies/mL, the algorithm recommended providing adherence support and repeat HIV VL testing within 3 to 4 months from the initial measurement. If the confirmatory HIV VL was again ≥1,000 copies/mL, ART modification for a new ART line was recommended; otherwise, no ART modification was required.

Adherence to ART was assessed at each clinical visit by self-reporting the number of missed ART doses in the last 4 days (ranging from none to four missed doses).

### Blood sampling

At OPC level, DBS was used to collect blood specimen for HIV VL testing. Whole blood was collected by venous puncture, and immediately after collection, 70 μL of whole blood was dropped using a calibrated pipette onto each of the five spots of two Munktell TFN paper cards (Ahlstrom-Munksjö, Bärenstein, Germany). The DBS cards were then left to dry on a rack at ambient temperature for a minimum of three hours, after which they were packed individually into a sealable plastic bag with three desiccants. The packed DBS cards were stored at ambient temperature until the transfer to the National Reference Laboratory of HIV Molecular Biology at the National Institute of Hygiene and Epidemiology (NIHE) in Hanoi (Vietnam). DBS were shipped once a week at ambient temperature, by postal service, directly from each OPC to the NIHE. However, some OPCs initially sent the DBS to an intermediate laboratory in the provincial capital which then transferred the DBS to Hanoi. This affected a limited number of OPCs for only a short time, and therefore a limited number of DBS.

### HIV viral load testing

HIV VL testing on DBS was performed at the National Reference Laboratory of HIV Molecular Biology at the NIHE, where the technique used has previously been evaluated. VL was measured following the manufacturer’s recommendations with the Abbott RealTime HIV-1 assay (Abbott Molecular, Des Plaines, IL, USA) which is CE-marked and WHO pre-qualified for DBS. Briefly, one spot was taken out of the card and placed in a tube containing 1.3 mL of lysis buffer and then incubated for 30 minutes at 55°C. The tubes were then loaded into the Abbott m2000sp platform for RNA extraction. The extraction products were transferred to the Abbott m2000rt machine in which RNA was reverted to cDNA and where amplification/detection took place. The technique’s lower limit of detection was 839 copies/mL.

### Statistical analysis

Baseline characteristics were described between PWID and non-PWID using median and interquartile range (IQR) for continuous variables and frequency and proportions for categorical variables. Comparisons were made using student’s t-test for the continuous variables and chi-2 test for categorical variables.

DBS coverage at 6, 12 and 24 months of ART was estimated as the proportion of patients still followed at these timepoints and in whom DBS had been collected, irrespective of DBS availability at previous timepoints. Patients were considered still followed if they attended a visit in the period ranging from 2 months prior to 4 months after the timepoint. Factors associated with DBS coverage were identified using mixed-effects logistic regression models to account for repeated measurements from the same patient.

Virological failure at 6, 12 and 24 months of ART was defined as having a VL ≥1,000 copies/mL on DBS collected during the period ranging from 2 months prior to 4 months after the timepoint. The confirmatory HIV VL was never considered in this definition and were analyzed separately. Factors associated with virological failure at 6, 12 and 24 months of ART were identified using mixed-effects logistic regression models. In a sensitivity analysis, we restricted the selection to the 24 months of ART timepoint.

In the statistical analyses described above, the period around the timepoints was considered to allow for variability in the timing of clinical visits and DBS collection, inherent to real-life conditions, and to describe what happens in real-life conditions.

To evaluate use of the HIV VL monitoring algorithm, the proportion of patients in virological failure for whom a confirmatory DBS was collected was measured. Patients without confirmatory DBS because they died, were transferred or lost to follow-up (LTFU) were not considered. Factors associated with the availability of a confirmatory DBS were investigated using mixed-effects logistic regression models.

The Vietnamese population consists in a large number of ethnic groups, not necessarily sharing the same language, the Kinh ethnic group being the main ethnic group in the country. In our analyses, we considered the ethnic group (Kinh versus the other ethnic minorities) to see if it impacted care and our health outcomes.

In all analyses, late visit attendance was defined as being at least seven days late at a visit, at least once, within the 3 months preceding the evaluation timepoint. An alternative definition considered a delay of ten days. Non-adherence was self-reported and defined as having missed at least one drug intake in the last four days, to at least one of the attended visits within the 3 months preceding the evaluation timepoint. Body mass index (BMI) was categorized using the threshold defining severe to moderate underweight, underweight and overweight (17, 18.5 and 25, respectively). WHO stage within the 3 months preceding the evaluation timepoint was categorized as asymptomatic HIV infection with some clinical manifestations (Stage 1–2), moderate symptomatic stage (Stage 3) or AIDS stage (Stage 4).

In all analyses, factors associated with the outcomes with a p-value <0.20 in the univariate analysis were considered in the multivariate analysis. In the multivariate analyses, a backward stepwise procedure was used to identify factors independently associated with the outcome. However, PWID status, was added to the models irrespective of its p-value in the univariate analysis. A p-value <0.05 was considered statistically significant. All analyses were performed using Stata software (Stata Corp., College Station, TX, USA).

### Ethical considerations

The study was reviewed and approved by the Institutional Review Board at the NIHE in Vietnam (02/QD-VSDTTU) and Institut Pasteur in France (2016-10/IRB/1). The protocol was submitted on the clinicaltrials.gov website (NCT03249493). All participants gave their written informed consent before the enrolling. Data processing was authorized by the French legal authority CNIL (DR-2017-046).

## Results

From June 2017 to April 2018, 578 HIV-infected adults who initiated ART were enrolled in the cohort. All participants initiated ART with a combination of Lamivudine, Tenofovir and Efavirenz (3TC-TDF-EFV).

Overall, 261/578 (45.2%) patients were former or current PWID (Table 1). As previously shown [8], the proportion of men was significantly higher in PWID, and PWID were significantly older and less often followed in urban settings.

**Table 1:** Baseline characteristics of PWID and non-PWID.

	PWID (n = 261)	non-PWID (n = 317)	P
Male gender, n (%)	254 (97.3)	174 (54.9)	<0.001
Age at ART initiation (years), median (IQR)	35 (30–40)	32 (27–38)	<0.001
Urban care setting, n (%)	80 (30.6)	155 (48.9)	<0.001

PWID: people who inject drugs; ART: antiretroviral therapy; IQR: inter quartile range

### DBS coverage

To globally assess routine HIV VL monitoring, we first quantified DBS coverage. In patients who were still followed, DBS coverage increased from 401/537 (74.7%) at 6 months of ART to 414/493 (84.0%) and 369/445 (82.9%) at 12 and 24 months of ART, respectively (Tables 2 and 3; p = 0.001). The increase in DBS coverage was also evidenced when time since the beginning of the study (i.e. since DBS availability) was investigated (p<0.001). This remained true in multivariate analysis. Disparities in DBS coverage were observed between provinces (p<0.001), with Thanh Hoa presenting a significantly lower DBS coverage. DBS coverage was also found to be significantly lower in rural settings after adjusting for province (p = 0.025). DBS coverage tended to be lower in patients who were late by seven days or more, on at least one occasion, within the 3 months preceding the timepoint (p = 0.052). After adjusting for the timepoint and late visit attendance, DBS coverage was lower in those in WHO stage 4 (p<0.001). On the other hand, when, PWID status was forced in the multivariate analysis, DBS coverage was not different in PWID as compared to non-PWID (p = 0.72). All these results were unchanged when the timepoint was considered in the multivariate analysis, instead of the time since the beginning of the study.

**Table 2:** Patient status at 6, 12 and 24 months of ART.

	6 months	12 months	24 months
Status at the timepoint, n			
Still followed	537	493	445
Dead	14	27	38
Transfer-out	11	21	43
Lost to follow-up	16	37	52
DBS coverage^1^, n (%)	401^2^ (74.7)	414^2^ (84.0)	369^2^ (82.9)
Virological evaluation^3^, n (%)			
<1,000 copies/mL	314 (84.2)	349 (85.8)	340 (93.4)
≥1,000 copies/mL	59 (15.8)	58 (14.2)	24 (6.6)
Confirmatory DBS, n (%)			
Yes	15 (25.4)	36 (62.1)	14 (58.3)
No, not collected	38 (64.4)	14 (24.1)	5 (20.8)
No, death	2 (3.4)	3 (5.2)	4 (16.7)
No, lost to follow-up	1 (1.7)	4 (6.9)	1 (4.2)
No, Transfer-out	3 (5.1)	1 (1.7)	-

DBS: dried blood spot

^1^Only DBS collected within the interval 2 months prior and 4 months after the time point were considered, proportions are estimated as the ratio between the number of DBS collected divided by the number of patients still followed.

^2^At 6 months, four patients had their DBS lost at some point, with no replacement; at 12 months, seven patients had their DBS lost at some point, and two patients had new DBS collected to replace them; at 24 months, one patient had his DBS lost at some point and a new DBS was collected to replace it.

^3^Some DBS were lost and therefore not tested (as described before) and others were discarded as they showed signs of humidity making the HIV viral load result uncertain (24, 2 and 5 DBS discarded at 6, 12 and 24 months of ART, respectively)

**Table 3:** Factors associated with DBS coverage (mixed-effects logistic regression).

	N	N (%) with DBS	Crude OR (95% CI)	P	Adj. OR (95% CI)	p
Timepoint				<0.001		
6 months	537	401 (74.7)	1			
12 months	493	414 (84.0)	1.95 (1.37–2.78)			
24 months	445	369 (82.9)	1.68 (1.17–2.42)			
Time since DBS availability				<0.001		<0.001
≤9 months	191	111 (58.1)	1		1	
10–18 months	735	613 (83.4)	4.07 (2.64–6.27)		3.05 (2.01–4.64)	
>19 months	549	460 (83.8)	4.09 (2.61–6.41)		3.05 (1.97–4.71)	
PWID				0.63		0.72
No	830	661 (79.6)	1		1	
Yes	645	523 (81.1)	1.10 (0.74–1.64)		0.94 (0.66–1.34)	
Gender				0.11		
Male	1069	846 (79.1)	1			
Female	406	338 (83.2)	1.45 (0.92–2.27)			
Age at ART initiation (years)				0.51		
≤29	481	384 (79.8)	1.14 (0.73–1.79)			
30–39	638	504 (79.0)	1			
40–59	296	244 (82.4)	1.36 (0.80–2.33)			
≥50	60	52 (86.7)	1.93 (0.65–5.70)			
Current WHO stage				0.031		<0.001
1–2	1398	1127 (80.6)	1		1	
3	59	47 (79.7)	0.79 (0.34–1.82)		0.55 (0.24–1.24)	
4	18	10 (55.6)	0.17 (0.04–0.64)		0.08 (0.02–0.31)	
Current BMI level				0.39		
<17.5	116	91 (78.4)	0.78 (0.40–1.54)			
17.5–20.0	495	389 (78.6)	0.77 (0.53–1.14)			
20.0–25.0	830	680 (81.9)	1			
≥25.0	34	24 (70.6)	0.48 (0.15–1.51)			
Rural care setting				0.11		0.025
No	592	490 (82.8)	1		1	
Yes	883	694 (78.6)	0.72 (0.48–1.07)		0.64 (0.44–0.95)	
Kinh ethnicity				0.74		
No	866	703 (81.2)	1			
Yes	530	417 (78.7)	0.85 (0.56–1.29)			
Unknown	79	64 (81.0)	1.02 (0.42–2.50)			
Late visit attendance by 7 days^1^				0.045		0.052
No	1286	1047 (81.4)	1		1	
Yes	189	137 (72.5)	0.63 (0.40–0.99)		0.65 (0.42–1.00)	
Self-reported non adherence^2^				0.64		
No	1334	1069 (80.1)	1			
yes	141	115 (81.6)	0.87 (0.49–1.56)			
Province				<0.001		<0.001
Lai Chau	135	119 (88.1)	0.20 (0.07–0.58)		0.24 (0.08–0.74)	
Lao Cai	127	105 (82.7)	0.12 (0.04–0.35)		0.13 (0.04–0.39)	
Phu Tho	202	196 (97.0)	1		1	
Thai Nguyen	183	167 (91.3)	0.30 (0.10–0.87)		0.24 (0.08–0.73)	
Thanh Hoa	609	405 (66.5)	0.04 (0.02–0.11)		0.04 (0.02–0.11)	
Yen Bai	219	192 (87.7)	0.19 (0.07–0.53)		0.20 (0.07–0.58)	

DBS: dried blood spot; OR: odds ratio; CI: confidence interval; PWID: people who inject drugs; ART: antiretroviral therapy; WHO: World Health Organization; BMI: body mass index

^1^Being late by at least seven days or more within the last 3 months preceding the evaluation time.

^2^Reporting having missed at least one dose of ART within the 4 days preceding the clinical visits on at least one occasion within the last 3 months preceding the evaluation time.

In an alternative multivariate analysis where late visit attendance by ten days or more was considered, being late was significantly associated with lower DBS coverage (p = 0.023), the effects of the other factors being unchanged.

### Virological evaluation

Out of the 1,184 DBS collected at 6, 12 and 24 months of ART, 9 (0.8%) were lost and never replaced and 31 (2.6%) were discarded from the analysis as the HIV VL result was undetectable but desiccants were showing signs of humidity or were absent, making the result questionable. The number of DBS discarded was 24, 2 and 5 at 6, 12 and 24 months of ART, respectively. Therefore, 373/537 (69.4%), 407/493 (82.6%) and 364/445 (81.8%) patients were virologically evaluated at 6, 12 and 24 months of ART, respectively.

The proportion of patients in virological failure, defined at the threshold of 1,000 copies/mL decreased with time from 15.8% (59/373) at 6 months of ART to 6.6% (24/364) at 24 months of ART (Table 4, p<0.001; see also S1 Table). In multivariate analysis, the proportion of patients in failure remained significantly larger in PWID (p = 0.001). In multivariate analysis, late clinical visit attendance by seven days or more in the 3 months preceding the evaluation, as well as self-declared sub-optimal adherence within the 3 months preceding the evaluation were both independently associated with increased risk of failure (p<0.001 and p = 0.001, respectively; Table 4). Patients in clinical stage 4 were also at increased risk of failure (p = 0.005), but only 10 patients were in stage 4. Even after adjusting for other factors, the rate of virological failure differed between provinces (p<0.001).

**Table 4:** Factors associated with virological failure defined as a viral load ≥1,000 copies/mL (mixed-effects logistic regression model).

	N	Virological failure	Crude OR (95% CI)	P	Adj. OR (95% CI)	P
Timepoint				<0.001		<0.001
6 months	373	59 (15.8)	1		1	
12 months	407	58 (14.2)	0.90 (0.52–1.57)		0.87 (0.57–1.31)	
24 months	364	24 (6.6)	0.24 (0.12–0.49)		0.35 (0.21–0.60)	
PWID				0.005		0.001
No	630	59 (9.4)	1		1	
Yes	514	82 (15.9)	3.00 (1.39–6.47)		1.89 (1.29–2.77)	
Gender				0.004		
Male	816	116 (14.2)	1			
Female	328	25 (7.6)	0.23 (0.08–0.63)			
Age at ART initiation (years)				0.66		
≤29	370	40 (10.8)	0.74 (0.31–1.81)			
30–39	489	61 (12.5)	1			
40–49	236	34 (14.4)	1.46 (0.55–3.87)			
≥50	49	6 (12.2)	0.92 (0.14–6.13)			
WHO stage				0.025		0.005
1–2	1091	128 (11.7)	1		1	
3	43	8 (18.6)	1.87 (0.45–7.77)		1.48 (0.61–3.54)	
4	10	5 (50.0)	39.51 (2.45–637.75)		9.18 (2.35–35.83)	
Current BMI level				0.49		
<17.5	90	14 (15.6)	1.83 (0.57–5.84)			
17.5–20.0	377	50 (13.3)	1.41 (0.70–2.86)			
20.0–25.0	652	74 (11.3)	1			
≥25.0	25	3 (12.0)	3.22 (0.40–25.56)			
Rural care setting				0.56		
No	469	55 (11.7)	1			
Yes	675	86 (12.7)	1.25 (0.58–2.69)			
Kinh ethnicity				0.64		
No	672	88 (13.1)	1			
Yes	410	47 (11.5)	0.86 (0.39–1.90)			
Unknown	62	6 (9.7)	0.42 (0.07–2.67)			
Distance to care site				0.21		
<10 km	306	31 (10.1)	1			
10 to 30 km	388	56 (14.4)	1.83 (0.70–4.79)			
>30 km	292	35 (12.0)	1.16 (0.41–3.29)			
Drug treatment center	75	4 (5.3)	0.27 (0.03–2.07)			
Unknown	83	15 (18.1)	2.96 (0.70–12.62)			
Sedentary activity				0.08		
No	255	19 (7.4)	1			
Yes	795	104 (13.1)	2.41 (0.89–6.48)			
Unknown	94	18 (19.5)	4.94 (1.13–21.57)			
Late visit attendance by 7 days^1^				<0.001		<0.001
No	1009	106 (10.5)	1		1	
Yes	135	35 (25.9)	5.94 (2.67–13.21)		2.60 (1.60–4.21)	
Self-reported non adherence^2^				0.023		0.001
No	1030	116 (11.3)	1		1	
Yes	114	25 (21.9)	2.66 (1.14–6.22)		2.69 (1.48–4.90)	
Province				0.028		<0.001
Lai Chau	118	16 (13.6)	2.21 (0.52–9.45)		2.24 (1.04–4.85)	
Lao Cai	105	15 (14.3)	2.18 (0.49–9.69)		1.75 (0.78–3.94)	
Phu Tho	195	17 (8.7)	1		1	
Thai Nguyen	167	8 (4.8)	0.25 (0.05–1.40)		1.02 (0.40–2.58)	
Thanh Hoa	370	66 (17.8)	3.02 (0.99–9.27)		4.18 (2.16–8.08)	
Yen Bai	189	19 (10.0)	1.05 (0.27–4.05)		1.98 (0.93–4.23)	

OR: odds ratio; CI: confidence interval; PWID: people who inject drugs; ART: antiretroviral therapy; WHO: World Health Organization; BMI: body mass index

^1^being late by at least seven days or more within the last 3 months preceding the evaluation time.

^2^Reporting having missed at least one dose of ART within the 4 days preceding the clinical visit on at least one occasion within the last 3 months preceding the evaluation time

In an alternative multivariate analysis considering late visit attendance by ten days or more, being late showed an even stronger association (adjusted OR (95% CI): 3.49 (2.11–5.78); p<0.001) while the effects of the other factors were unchanged (data not shown).

Restricting the analysis to the 24 months of ART timepoint, the only two factors that remained independently and significantly associated with higher risk of virological failure were PWID status and late visit attendance (data not shown).

### Confirmation of virological failure

According to the national algorithm, patients in virological failure should receive adherence support and have another DBS collected within 3 to 4 months following the initial DBS. A confirmatory DBS was collected in 15/59 (25.4%), 36/58 (62.1%) and 14/24 (58.3%) patients who were in virological failure at 6, 12 and 24 months of ART, respectively (Table 2). Excluding patients who died, were transferred to another OPC or LTFU within the 6 months following the initial DBS collection the only factor significantly associated with the presence of a confirmatory DBS was the timepoint (Table 5).

**Table 5:** Factors associated with DBS availability to confirm virological failure (mixed-effects logistic regression).

	N	N (%) with DBS	Crude OR (95% CI)	P
Timepoint				<0.001
6 months	53	15 (28.3)	1	
12 months	50	36 (72.0)	6.51 (2.76–15.38)	
24 months	19	14 (73.7)	7.09 (2.17–23.15)	
PWID				0.18
No	50	23 (46.0)	1	
Yes	72	42 (58.3)	1.64 (0.79–3.40)	
Gender				0.58
Male	98	51 (52.0)	1	
Female	24	14 (58.3)	1.29 (0.52–3.18)	
Age at ART initiation (years)				0.050
≤29	33	23 (69.7)	3.03 (1.20–7.66)	
30–39	51	22 (43.1)	1	
40–59	32	15 (46.8)	1.16 (0.48–2.83)	
≥50	6	5 (83.1)	6.59 (0.72–60.53)	
Current WHO stage				0.82
1–2	111	59 (54.0)	1	
3	6	3 (50.0)	0.85 (0.16–4.40)	
4	5	2 (40.0)	0.57 (0.10–3.52)	
Current BMI level				0.06
<17.5	6	2 (33.3)	0.62 (0.11–3.63)	
17.5–20.0	49	32 (65.3)	2.34 (1.11–5.02)	
20.0–25.0	65	29 (44.6)	1	
≥25.0	2	2 (100.0)	*Not estimated*	
Rural care setting				0.24
No	51	24 (47.1)	1	
Yes	71	41 (57.7)	1.54 (0.75–3.17)	
Kinh ethnicity				0.74
No	78	40 (51.3)	1	
Yes	38	21 (55.3)	1.17 (0.54–2.56)	
Unknown	6	4 (66.7)	1.90 (0.33–10.99)	
Late visit attendance by 7 days^1^				0.42
No	99	51 (51.5)	1	
Yes	23	17 (60.9)	1.46 (0.58–3.69)	
Self-reported non adherence^2^				0.51
No	102	53 (52.0)	1	
yes	20	12 (60.0)	1.39 (0.52–3.68)	
Province				0.18
Lai Chau	14	9 (64.3)	0.65 (0.13–3.19)	
Lao Cai	13	9 (68.2)	0.82 (0.16–4.23)	
Phu Tho	15	11 (73.3)	1	
Thai Nguyen	8	4 (50.0)	0.36 (0.06–2.20)	
Thanh Hoa	58	24 (41.4)	0.26 (0.07–0.90)	
Yen Bai	14	8 (57.1)	0.49 (0.10–2.31)	

DBS: dried blood spot; OR: odds ratio; CI: confidence interval; PWID: people who inject drugs; ART: antiretroviral therapy; WHO: World Health Organization; BMI: body mass index

^1^being late by at least seven days or more within the last 3 months preceding the evaluation time.

^2^Reporting having missed at least one dose of ART within the 4 days preceding the clinical visit on at least one occasion within the last 3 months preceding the evaluation time

In patients with a confirmatory DBS, 11/15 (73.3%), 12/36 (33.3%) and 6/14 (42.8%) were in confirmed virological failure at 6, 12 and 24 months of ART, respectively. Overall, 52 patients were identified in virological failure during follow-up and only one (1.9%) was switched to second-line ART.

## Discussion

The MOVIDA study aimed at increasing access to HIV VL monitoring in remote settings using DBS, and to evaluate different aspects of HIV VL monitoring. Prior to the MOVIDA study, no routine HIV VL monitoring was implemented in the six provinces. All clinical and laboratory teams were trained to use DBS for routine HIV VL monitoring. Despite that training, regular contacts with the clinical teams for monitoring reasons (which would not happen in real-life conditions), and simple procedures for sample collection, storage and transfer, DBS coverage was not 100%. However, DBS coverage increased with time to reach 83.8% and 81.8% at 12 and 24 months of ART, respectively. Sub-optimal coverage was also reported by others, including *Médecins Sans Frontières* in a 2016 report where, despite support, HIV VL coverage ranged from 32% to 91% [12, 13]. We also noted that the number of DBS that were lost decreased with time, as did the number of DBS incorrectly maintained and that showed signs of humidity. This suggests that appropriation of the tool and good integration into routine care necessitates time and training.

In multivariate analysis, disparities were observed between provinces in terms of DBS coverage. This probably illustrates differences between clinical sites, but limited sample sizes in each OPC did not allow investigations at that level. Differences between clinical sites could be explained by the involvement of clinical staff, management at the OPC or workload. We did not, however, record the total number of patients followed at each site to investigate its potential impact. It is also likely that, in some OPCs, staff turnover led to lower DBS coverage.

Interestingly, late clinical visit attendance was associated with lower DBS coverage. Patients’ attitude towards their care could have influenced the health workers’ decision to collect a DBS or not. Patients in WHO stage 4 were less likely to have DBS collected, even though they would greatly benefit from HIV VL monitoring. Although this should be confirmed by a dedicated socio-anthropological qualitative study, one could wonder if these results reflect prejudice from clinical staff against patients who show signs of failure and are believed to be non-adherent to ART. On the other hand, while it has been shown that PWID often experience lower and delayed access to care [14, 15], their DBS coverage was not reduced.

Previous studies reported that workload and complexity of the process to obtain results limited access to HIV viral load monitoring [16]. In Vietnam, qualitative studies interviewing clinical staff on workload, integration of DBS collection and HIV VL testing in routine care could help understand the reasons for sub-optimal DBS coverage, which would be useful to implement routine HIV VL monitoring successfully. Empowering patients to self-manage their disease by requesting HIV VL testing is also crucial to boost both the clinical staff and patient motivation to create demand for HIV VL testing [13, 17].

At some point, some patients saw their DBS lost. Unfortunately, in most cases, the clinical team did not realize that no HIV VL result was returned to these patients, and therefore no new samples were collected. A small proportion of DBS (2.6%) showed quality defects. The NIHE laboratory informed the OPC about the defect observed but this did not translate into a request for new DBS to be collected. Consequently, patients never received their HIV VL result. This is worrying as it could create distrust in the care system and ultimately affect their adherence to ART but also compromise patient outcomes. Therefore, these gaps in DBS tracking should be addressed to improve the overall HIV monitoring system.

The proportion of patients in virological failure significantly decreased from 15.8% at 6 months of ART to 6.6% at 24 months of ART. The decrease in the proportion of patients in virological failure is likely partly explained by the healthy survivor effect. Indeed, more adherent patients are more likely to remain in care, hence leading to lower proportion of patients with high HIV VL. The result at 24 months of ART was consistent with previous cross-sectional surveys in Vietnam which showed >90% success in patients on ART for >24 months [18, 19].

PWID were at increased risk of virological failure. This was also evidenced in an earlier study conducted in Hanoi [20]. It has been shown that PWID still suffer from stigmatization and have lower access to health care [10, 11, 21]. Although this result was adjusted for late visit attendance and self-reported adherence, higher risk of virological failure in PWID may be due to lower compliance with ART. Indeed, marginalization and social isolation impeded adherence in PWID [22]. As one would expect, late visit attendance was associated with virological failure, as was self-declared non-adherence. These two factors, associated with virological failure, clearly identify patients for whom ART adherence is challenging. Efforts should therefore be made to identify the issues patients face so that specific actions can be taken to improve their virological outcomes. For example, limiting the number of visits to the clinical site through ART prescription for a longer time or through home visits could be beneficial in some patients. Previous studies identified that late ART initiation was associated with virological failure [23]. The limited number of CD4 cell measurements at baseline and the low number of patients in WHO stages 3–4 [8], probably due to limited diagnostic capacities, did not allow us to investigate this condition properly.

National guidelines, inspired by WHO guidelines, recommend adherence intervention followed by a new HIV VL measurement to confirm or invalidate virological failure. The proportion of patients in failure, for whom a confirmatory DBS was collected, improved with time from 25.4% at 6 months of ART to 60% at 12 and 24 months of ART. Our results are consistent with what was observed by *Médecins Sans Frontières* in its programs [13]. Again, this result suggests that time is necessary before the appropriation of DBS use.

Worryingly, only 1 out of 52 patients in failure was switched to second line. This figure, consistent with previous findings [24], shows that efforts must be made to improve access to second-line ART.

This study has some limitations. The population enrolled was essentially men, partly because 45% of our study population consisted of PWID. However, the over-representation of men in Vietnamese HIV programs has been reported by others [18–20, 25] and is representative of the country’s situation. Although the study was performed in real-life conditions, it included on-site monitoring and reminders to clinical teams. This could have impacted and improved DBS coverage, but it is unlikely that it influenced the virological results and associated factors. At ART initiation few patients (28%) had their CD4 count measured, essentially due to implementation of the test and treat strategy in Vietnam. Indeed, CD4 measurements are no longer necessary to identify those who need ART as all PLHIV should receive it. This trend towards lower CD4 measuring has also been reported in other settings [26, 27]. CD4 measurement remains important for patients with CD4 <100 cells/mm^3^, however, who should have faster ART initiation and compulsory screening for other diseases such as Cryptococcus and Tuberculosis. Consequently, it did not allow us to describe the extent of late ART initiation. Some bias might influence the interpretation of VL suppression. For instance, adherence should be interpreted with caution because it was self-reported and subject to desirability bias. However, the fact that those who self-reported being non-adherent were also more often in virological failure suggests that the information provided is very relevant.

## Conclusions

Despite training and simple procedures, DBS coverage was not perfect, suggesting the need to invest in close management for effective routine HIV VL monitoring. PWID were more at risk of failure, as were late presenters and patients who reported not being fully adherent. Interventions targeting these patients are needed to improve their outcomes.

## Supporting information

S1 Table Description of the 59 failures at 6 months of ART (M6) and their outcomes at 12 months of ART (M12). ART: antiretroviral therapy; VL: viral load; LTFU: lost to follow-up *these events occurred before a blood sample for confirmatory VL testing at M6 was collected **this event occurred after the confirmatory VL at M6 was measured but before a blood sample for VL testing at M12 was collected.
(DOCX)

## References

[1] HIV data and statistics [Internet]. [cited 2021 Jul 13]. Available from: https://www.who.int/teams/immunization-vaccines-and-biologicals/diseases/tick-borne-encephalitis/hiv

[2] Consolidated Guidelines on the Use of Antiretroviral Drugs for Treating and Preventing HIV Infection: Recommendations for a Public Health Approach [Internet]. 2nd ed. Geneva: World Health Organization; 2016 [cited 2021 Jul 13]. (WHO Guidelines Approved by the Guidelines Review Committee). Available from: http://www.ncbi.nlm.nih.gov/books/NBK374294/

[3] Monleau M, Aghokeng AF, Eymard-Duvernay S, Dagnra A, Kania D, Ngo-Giang-Huong N, et al. Field evaluation of dried blood spots for routine HIV-1 viral load and drug resistance monitoring in patients receiving antiretroviral therapy in Africa and Asia. J Clin Microbiol. 2014 Feb;52(2):578–86. doi: 10.1128/JCM.02860-13 24478491 PMC3911301

[4] Taieb F, Tram TH, Ho HT, Pham VA, Nguyen L, Pham BH, et al. Evaluation of Two Techniques for Viral Load Monitoring Using Dried Blood Spot in Routine Practice in Vietnam (French National Agency for AIDS and Hepatitis Research 12338). Open Forum Infect Dis. 2016 Sep;3(3):ofw142. doi: 10.1093/ofid/ofw142 27704001 PMC5047401

[5] Taieb F, Tran Hong T, Ho HT, Nguyen Thanh B, Pham Phuong T, Viet Ta D, et al. First field evaluation of the optimized CE marked Abbott protocol for HIV RNA testing on dried blood spot in a routine clinical setting in Vietnam. PloS One. 2018;13(2):e0191920. doi: 10.1371/journal.pone.0191920 29425216 PMC5806875

[6] Zeh C, Ndiege K, Inzaule S, Achieng R, Williamson J, Chih-Wei Chang J, et al. Evaluation of the performance of Abbott m2000 and Roche COBAS Ampliprep/COBAS Taqman assays for HIV-1 viral load determination using dried blood spots and dried plasma spots in Kenya. PloS One. 2017;12(6):e0179316. doi: 10.1371/journal.pone.0179316 28622370 PMC5473550

[7] Boillot F, Serrano L, Muwonga J, Kabuayi JP, Kambale A, Mutaka F, et al. Implementation and Operational Research: Programmatic Feasibility of Dried Blood Spots for the Virological Follow-up of Patients on Antiretroviral Treatment in Nord Kivu, Democratic Republic of the Congo. J Acquir Immune Defic Syndr 1999. 2016 Jan 1;71(1):e9–15. doi: 10.1097/QAI.0000000000000844 26413848 PMC4679362

[8] Nguyen TA, Tran TH, Nguyen BT, Pham TTP, Hong Le NT, Ta DV, et al. Feasibility of dried blood spots for HIV viral load monitoring in decentralized area in North Vietnam in a test-and-treat era, the MOVIDA project. PloS One. 2020;15(4):e0230968. doi: 10.1371/journal.pone.0230968 32271796 PMC7145146

[9] Viet Nam [Internet]. [cited 2022 Apr 25]. Available from: https://www.unaids.org/en/regionscountries/countries/vietnam

[10] Van Nguyen H, Nguyen HLT, Mai HT, Le HQ, Tran BX, Hoang CD, et al. Stigmatization among methadone maintenance treatment patients in mountainous areas in northern Vietnam. Harm Reduct J. 2017 Dec;14(1):1. doi: 10.1186/s12954-016-0127-9 28056990 PMC5217586

[11] Thanh DC, Moland KM, Fylkesnes K. Persisting stigma reduces the utilisation of HIV-related care and support services in Viet Nam. BMC Health Serv Res. 2012 Dec;12(1):428. doi: 10.1186/1472-6963-12-428 23176584 PMC3549738

[12] Kubheka SE, Archary M, Naidu KK. HIV viral load testing coverage and timeliness after implementation of the wellness anniversary in a paediatric and adolescent HIV clinic in KwaZulu-Natal, South Africa. South Afr J HIV Med [Internet]. 2020 Feb 3 [cited 2021 Jul 21];21(1). Available from: http://www.sajhivmed.org.za/index.php/HIVMED/article/view/1016 10.4102/sajhivmed.v21i1.1016 PMC7059249 32158554

[13] Making Viral Load Routine [Internet]. Médecins Sans Frontières Access Campaign. [cited 2021 Jul 21]. Available from: https://msfaccess.org/making-viral-load-routine

[14] Lan CW, Lin C, Thanh DC, Li L. Drug-related stigma and access to care among people who inject drugs in Vietnam. Drug Alcohol Rev. 2018;37(3):333–9. doi: 10.1111/dar.12589 28762584 PMC5794669

[15] Rangarajan S, Tram HNB, Todd CS, Thinh T, Hung V, Hieu PT, et al. Risk Factors for Delayed Entrance into Care after Diagnosis among Patients with Late-Stage HIV Disease in Southern Vietnam. Clark JL, editor. PLoS ONE. 2014 Oct 16;9(10):e108939. doi: 10.1371/journal.pone.0108939 25330196 PMC4199603

[16] Rutstein SE, Golin CE, Wheeler SB, Kamwendo D, Hosseinipour MC, Weinberger M, et al. On the front line of HIV virological monitoring: barriers and facilitators from a provider perspective in resource-limited settings. AIDS Care. 2016;28(1):1–10. doi: 10.1080/09540121.2015.1058896 26278724 PMC4834050

[17] Peter T, Ellenberger D, Kim AA, Boeras D, Messele T, Roberts T, et al. Early antiretroviral therapy initiation: access and equity of viral load testing for HIV treatment monitoring. Lancet Infect Dis. 2017 Jan;17(1):e26–9. doi: 10.1016/S1473-3099(16)30212-2 27773596 PMC5745573

[18] Rangarajan S, Colby DJ, Giang LT, Bui DD, Hung Nguyen H, Tou PB, et al. Factors associated with HIV viral load suppression on antiretroviral therapy in Vietnam. J Virus Erad. 2016 Apr;2(2):94–101. 27482442 10.1016/S2055-6640(20)30466-0 PMC4965252

[19] Dat VQ, Duong BD, Nhan DT, Hai NH, Anh NTL, Thu HHK, et al. Viral load suppression and acquired HIV drug resistance in adults receiving antiretroviral therapy in Viet Nam: results from a nationally representative survey. West Pac Surveill Response J. 2018 Sep 30;9(3):16–24. doi: 10.5365/wpsar.2018.9.1.008 30377546 PMC6194224

[20] Tanuma J, Matsumoto S, Haneuse S, Cuong DD, Vu TV, Thuy PTT, et al. Long-term viral suppression and immune recovery during first-line antiretroviral therapy: a study of an HIV-infected adult cohort in Hanoi, Vietnam. J Int AIDS Soc. 2017 Dec;20(4):e25030. doi: 10.1002/jia2.25030 29211347 PMC5810334

[21] Heath AJ, Kerr T, Ti L, Kaplan K, Suwannawong P, Wood E, et al. Healthcare avoidance by people who inject drugs in Bangkok, Thailand. J Public Health. 2016 Sep;38(3):e301–8. doi: 10.1093/pubmed/fdv143 26491067 PMC5072167

[22] Davis A, McCrimmon T, Dasgupta A, Gilbert L, Terlikbayeva A, Hunt T, et al. Individual, social, and structural factors affecting antiretroviral therapy adherence among HIV-positive people who inject drugs in Kazakhstan. Int J Drug Policy. 2018 Dec;62:43–50. doi: 10.1016/j.drugpo.2018.08.014 30359872 PMC6279490

[23] d’Amour Ndahimana J, Riedel DJ, Mwumvaneza M, Sebuhoro D, Uwimbabazi JC, Kubwimana M, et al. Drug resistance mutations after the first 12 months on antiretroviral therapy and determinants of virological failure in Rwanda. Trop Med Int Health. 2016;21(7):928–35. doi: 10.1111/tmi.12717 27125473

[24] Madec Y, Leroy S, Rey-Cuille MA, Huber F, Calmy A. Persistent Difficulties in Switching to Second-Line ART in Sub-Saharan Africa—A Systematic Review and Meta-Analysis. Graham SM, editor. PLoS ONE. 2013 Dec 23;8(12):e82724. doi: 10.1371/journal.pone.0082724 24376570 PMC3871158

[25] Tran DA, Shakeshaft A, Ngo AD, Mallitt KA, Wilson D, Doran C, et al. Determinants of antiretroviral therapy initiation and treatment outcomes for people living with HIV in Vietnam. HIV Clin Trials. 2013 Feb;14(1):21–33. doi: 10.1310/hct1401-21 23372112

[26] Ehrenkranz PD, Baptiste SL, Bygrave H, Ellman T, Doi N, Grimsrud A, Jet al. The missed potential of CD4 and viral load testing to improve clinical outcomes for people living with HIV in lower-resource settings. PLOS Med. 2019 May 29;16(5):e1002820. doi: 10.1371/journal.pmed.1002820 31141516 PMC6541248

[27] Zaniewski E, Dao Ostinelli CH, Chammartin F, Maxwell N, Davies M, Euvrard J, et al. Trends in CD4 and viral load testing 2005 to 2018: multi‐cohort study of people living with HIV in Southern Africa. J Int AIDS Soc [Internet]. 2020 Jul [cited 2021 Jul 22];23(7). Available from: https://onlinelibrary.wiley.com/doi/10.1002/jia2.25546 32640106 10.1002/jia2.25546 PMC7343336

