# Supplementary material for: Assessment of HIV viral load monitoring in remote settings in Vietnam - comparing people who inject drugs to the other patients
Source: PLoS One. 2023 Feb 21;18(2):e0281857. doi: 10.1371/journal.pone.0281857 (PMC9942987; doi:10.1371/journal.pone.0281857)
Supplement: S1 Table — ART: antiretroviral therapy; VL: viral load; LTFU: lost to follow-up *these events occurred before a blood sample for confirmatory VL testing at M6 was collected **this event occurred after the confirmatory VL at M6 was measured but before a blood sample for VL testing at M12 was collected. (DOCX) [file pone.0281857.s001.docx]

S1 Table: Description of the 59 failures at 6 months of ART (M6) and their outcomes at 12 months of ART (M12)

|  | Without confirmatory VL at M6 (N=44) | With confirmatory VL at M6 (N=15) |
| --- | --- | --- |
| **Outcome at M12**  VL <1000 copies/mL  VL>1000 copies/mL  In care, but no VL available  Death  LTFU  Transfer-out | 14  18  6  2*  3*  1* | 3  9  2  1**  -  - |

ART: antiretroviral therapy; VL: viral load; LTFU: lost to follow-up

*these events occurred before a blood sample for confirmatory VL testing at M6 was collected

**this event occurred after the confirmatory VL at M6 was measured but before a blood sample for VL testing at M12 was collected
